# The Anterior Prefrontal Cortex and the Hippocampus Are Negatively Correlated during False Memories

**DOI:** 10.3390/brainsci7010013

**Published:** 2017-01-23

**Authors:** Brittany M. Jeye, Jessica M. Karanian, Scott D. Slotnick

**Affiliations:** Department of Psychology, Boston College, Chestnut Hill, MA 02467, USA; jessica.karanian@bc.edu (J.M.K.); sd.slotnick@bc.edu (S.D.S.)

**Keywords:** false memory, hippocampus, anterior prefrontal cortex, dorsolateral prefrontal cortex, prefrontal cortex, fMRI

## Abstract

False memories commonly activate the anterior/dorsolateral prefrontal cortex (A/DLPFC) and the hippocampus. These regions are assumed to work in concert during false memories, which would predict a positive correlation between the magnitudes of activity in these regions across participants. However, the A/DLPFC may also inhibit the hippocampus, which would predict a negative correlation between the magnitudes of activity in these regions. In the present functional magnetic resonance imaging (fMRI) study, during encoding, participants viewed abstract shapes in the left or right visual field. During retrieval, participants classified each old shape as previously in the “left” or “right” visual field followed by an “unsure”–“sure”–“very sure” confidence rating. The contrast of left-hits and left-misses produced two activations in the hippocampus and three activations in the left A/DLPFC. For each participant, activity associated with false memories (right–“left”–“very sure” responses) from the two hippocampal regions was plotted as a function of activity in each A/DLPFC region. Across participants, for one region in the left anterior prefrontal cortex, there was a negative correlation between the magnitudes of activity in this region and the hippocampus. This suggests that the anterior prefrontal cortex might inhibit the hippocampus during false memories and that participants engage either the anterior prefrontal cortex or the hippocampus during false memories.

## 1. Introduction

The hippocampus has long been known to play a critical role in accurate long-term memories (i.e., true memories). There is also evidence that the hippocampus can be involved in the construction of false memories—memories for events that never occurred [1,2,3]. Previous functional magnetic resonance imaging (fMRI) studies have demonstrated that there can be overlapping neural activity in the hippocampus during true memory and false memory [4,5,6,7,8,9,10,11,12]. For example, in our recent spatial memory fMRI study [13], participants viewed abstract shapes in either the left or right visual field during the study phase. During the test phase, old shapes from the study phase were presented at fixation and participants identified whether each shape was previously presented in the “left” or “right” visual field and made an “unsure”–“sure”–“very sure” confidence rating. The same regions of the hippocampus were found to be associated with true memory for spatial location and false memory for spatial location. Such hippocampal activations are thought to reflect the binding of item information and context information during memory [14,15]. That is, during true memory, the hippocampus appears to bind item information with the correct context (e.g., the correct spatial location), and, during false memory, the hippocampus appears to bind item information with the incorrect context (e.g., the incorrect spatial location).

Like the hippocampus, the anterior/dorsolateral prefrontal cortex has also been associated with both true memory and false memory [3,16]. True memory and false memory activity in the left anterior/dorsolateral prefrontal cortex may reflect context memory [17,18]. Specifically, true memories can involve retrieval of the correct context and false memories can involve retrieval of the incorrect context [2]. The anterior/dorsolateral prefrontal cortex has also been associated with the subjective confidence during memory [19,20]. As true memories and false memories are often associated with high confidence, activity in the anterior/dorsolateral prefrontal cortex may also reflect this cognitive function.

The previous evidence indicates that the left anterior/dorsolateral prefrontal cortex and the hippocampus are associated with false memory. These regions are generally thought to work in concert during false memories, which would predict a positive correlation between the magnitudes of activity in these regions across participants. However, there is evidence that the anterior/dorsolateral prefrontal cortex may inhibit the hippocampus during retrieval, such as during motivated forgetting [21,22] and retrieval-induced forgetting [23]. If the anterior/dorsolateral prefrontal cortex inhibits the hippocampus during false memories, this would predict a negative correlation between the magnitudes of activity in these regions across participants.

In the current spatial memory fMRI study, to distinguish between the previous hypotheses, we evaluated the correlation between the magnitudes of activity in the left anterior/dorsolateral prefrontal cortex and the hippocampus during false memory. To anticipate the results, we found that the magnitude of false memory activity across participants was negatively correlated between these regions.

## 2. Materials and Methods

### 2.1. Participants

Sixteen right-handed Boston College students who had normal or corrected-to-normal vision and were native English speakers participated in the study (12 females, age range 22–28 years). The Boston College Institutional Review Board approved the protocol (identification code: 10.008, initial approval date: 9 December 2009) and informed consent was obtained prior to the behavioral training session. Each participant was compensated $10 for the behavioral training session and $25 per hour for the fMRI session. The results of the current study are an extension of our recent fMRI study [13] (the same participants, paradigm, and analysis protocol was employed, but the present correlation analysis was independent of the previous analysis).

### 2.2. Stimulus Protocol

**Figure 1:**
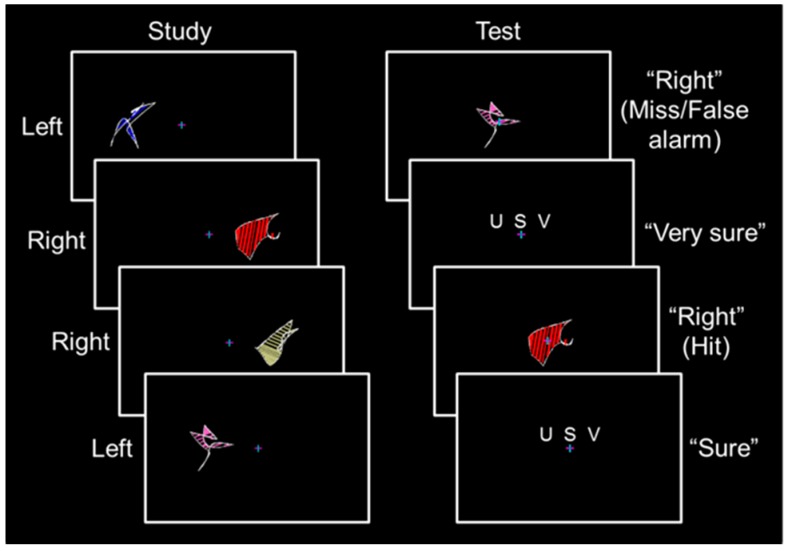
Stimulus protocol. **Left**, during the study phase, participants viewed abstract shapes to the left or right of fixation (labeled to the left). **Right**, during the test phase, old shapes were presented at fixation and participants classified each shape as previously on the “left” or “right” and made an “unsure”–“sure”–“very sure” confidence rating (possible responses and corresponding event types are shown to the right).

Participants completed a behavioral training session, which included a one-quarter length run and a full-length run, and seven to eight full-length runs during the fMRI session. During the study phase of each full-length run, 32 abstract shapes spanning 6.7° of visual angle were presented with their nearest edge 3.6° of visual angle from a central fixation cross (Figure 1, left; for information on shape construction, see [5]). Each shape was displayed for 2.5 s followed by a 0.5 s fixation period. An equal number of shapes were presented in the left and right visual fields. Participants were instructed to remember each shape and its spatial location while maintaining fixation. Shape sets were presented three times, with each shape set randomized and presented sequentially.

Before each test phase, an instruction screen was displayed for 8 s followed by a 2 s fixation period. During the test phase of each full-length run, the 32 shapes from encoding were presented in a random order at fixation for 3.0 s followed by a confidence rating reminder screen for 2.5 s and a fixation period of 0.5 to 4.5 s (Figure 1, right). Participants responded by pressing buttons with the fingers of their left hand to classify each shape as previously presented in the “left” or “right” visual field followed by a subsequent “unsure”–“sure”–“very sure” confidence rating. No more than three shapes of a given type were sequentially presented in the study phase or the test phase, shapes were never repeated across runs, and shape location (i.e., left and right) was counterbalanced across participants using a Latin square design.

### 2.3. Data Acquisition and Analysis

A Siemens 3 Tesla Trio Scanner (Siemens, Erlangen, Germany) with a 32-channel head coil was used to acquire imaging data. Anatomic images were acquired with a magnetization prepared rapid gradient echo sequence (repetition time = 30 ms, echo time = 3.3 ms, flip angle = 40°, field-of-view = 256 × 256 mm, acquisition matrix = 256 × 256, slices = 128, slice thickness = 1 mm; 1.33 × 1 × 1 mm resolution). Functional images were acquired with an echo planar imaging sequence (repetition time = 2000 ms, echo time = 30 ms, flip angle = 90°, field-of-view = 256 × 256 mm, acquisition matrix = 64 × 64, slices = 33, slice acquisition order = interleaved bottom-to-top, slice thickness = 4 mm, no gap; 4 mm isotropic resolution). BrainVoyager 20.0 (Brain Innovation B.V., Maastricht, The Netherlands) was used to conduct the analyses. Pre-processing of the functional images included motion correction, slice-time correction, and removal of temporal components below two cycles per run length (using a general linear model to remove low frequency Fourier basis sets). Voxels were resampled at 3 × 3 × 3 mm. To maximize spatial resolution, spatial smoothing was not conducted. Anatomic and functional images were transformed into Talairach space.

A random-effect general linear model analysis was conducted. Each event type was modeled based on its onset and the subsequent behavioral response (if a response was made). It was assumed that encoding trials and no-response trials had durations of 2.5 s. This produced the following event types: encoding location, accurate memory for spatial location, inaccurate memory for spatial location, no response, and a constant. The contrast of left–“right”–“very sure” and left–“left”–“very sure” was used to isolate activity associated with false memory for shapes in the “right” visual field and the contrast of right–“left”–“very sure” and right–“right”–“very sure” was used to isolate activity associated with false memory for shapes in the “left” visual field. These contrasts did not produce any activity in the hippocampus. Therefore, as neural activity for both true memory and false memory overlap in the hippocampus and the anterior/dorsolateral prefrontal cortex (see the Introduction), the contrast of left–“left” and left–“right” (i.e., true memory spatial location hits versus misses, collapsed over confidence) was used to isolate activity associated with spatial memory for shapes in the left visual field and the contrast of right–“right” and right–“left” was used to isolate activity associated with spatial memory for shapes in the right visual field. The true memory activations in the hippocampus and the anterior/dorsolateral prefrontal cortex served as regions of interest to extract and analyze false memory activity. For all contrasts, an individual voxel threshold of *p* < 0.001 was enforced, false discovery rate corrected for multiple comparisons to *p* < 0.05. Hippocampal activations within the medial temporal lobe were localized based on established anatomical distinctions [24,25,26,27]. All activations were localized on the mean group anatomic volume and each Talairach coordinate refers to the voxel with peak activity.

For each region of interest identified using the preceding analysis, event-related magnitudes were extracted from active voxels within a 5 mm cube (centered on the activation) from −2 to 12 s after stimulus onset (baseline corrected from −2 to 0 s). To ensure activation magnitudes were greater than or equal to baseline (corresponding to a lower boundary on neural firing of zero spikes per second), the minimum activation magnitude across all event types was subtracted from each activation timecourse [13,28]. As fMRI activity can be assumed to reflect the underlying neural activity [29], we subtracted the minimum activation magnitude in an effort to make the zero point in the magnitude of fMRI activity corresponding to the zero point of neural activity. Of importance, baseline correction resulted in a constant shift for all magnitudes in a given region and thus did not influence the correlation results. For each participant, the mean event-related magnitude of activity associated with false memories (i.e., high confidence false alarms) from 4 to 6 s after stimulus onset was used for analysis (i.e., the expected maximum amplitude of the hemodynamic response). Three participants (two males) who made no right–“left”–“very sure” responses were excluded from the fMRI analysis. The correlation results were Bonferroni corrected for multiple comparisons.

## 3. Results

**Figure 2:**
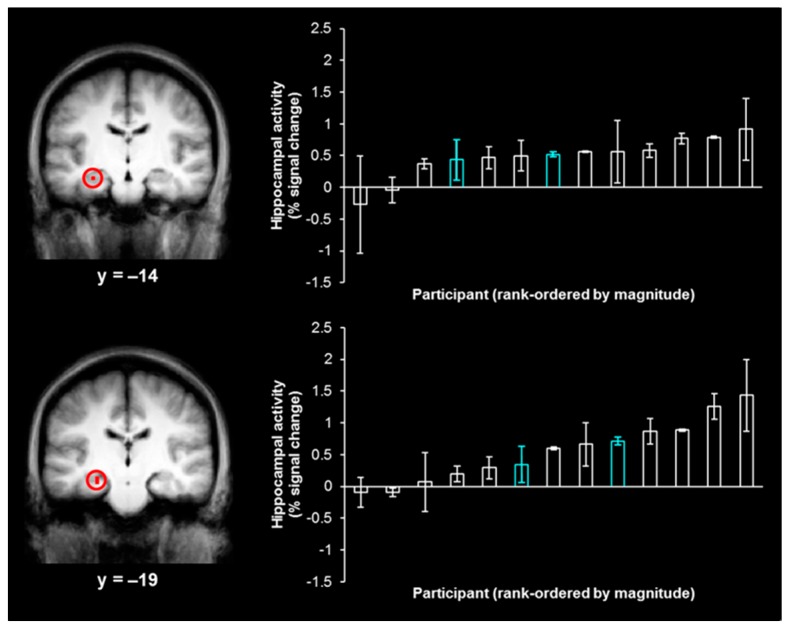
Hippocampal activity associated with true memory for items in the left visual field and the corresponding individual-participant magnitudes of hippocampal activity associated with false memory. **Left**, hippocampal activations associated with left-hits versus left-misses (circled in red; coronal views). **Right**, individual-participant magnitudes of activity (percent signal change) associated with false memories (right–“left”–“very sure” responses), rank ordered for the lowest to the highest magnitude of activity, corresponding to each hippocampal activation to the left (results from male participants are shown in blue).

Behavioral accuracy was at an intermediate level and did not differ for shapes previously presented in the left visual field (75.5% correct) and shapes previously presented in the right visual field (78.6% correct; *t*(15) < 1). The contrast of left-hits and left-misses, which was used to isolate activity associated with true memory, produced two activations in the hippocampus (Figure 2, left; *x* = −27, *y* = −14, *z* = −15, size = 54 mm^3^; *x* = −24, *y* = −19, *z* = −11, size = 27 mm^3^), while the analogous contrast of right-hits and right-misses did not produce any activity in the hippocampus, even at a reduced threshold of *p* < 0.01, uncorrected.

As the neural activity associated with true memory and false memory overlap in the hippocampus (see the Introduction), we extracted individual participant magnitudes of activity associated with false memory (e.g., right–“left”–“very sure” responses) from each hippocampal activation (Figure 2, right). As the activations were identified by contrasting left-hits and left-misses, only false memories for items in the “left” visual field (i.e., right–“left”–“very sure” responses) were expected to produce activity in these regions and were employed in the correlation analysis. Such false memories were no more likely to stem from shapes that were presented in the first or last 5 trials of each study phase than the middle 22 trials of each study phase (i.e., there was no evidence of primacy/recency effects; *χ*^2^ < 1). The range of values shown by the distribution of the magnitudes of activity associated with right–“left”–“very sure” responses demonstrates the variability in the magnitude of hippocampal activity during false memories across participants. This distribution suggests that there are some participants with hippocampal-dependent processing during false memories (associated with higher magnitudes of activity in this region) and some participants with hippocampal-independent processing during false memories (associated with lower magnitudes of activity in this region).

**Figure 3:**
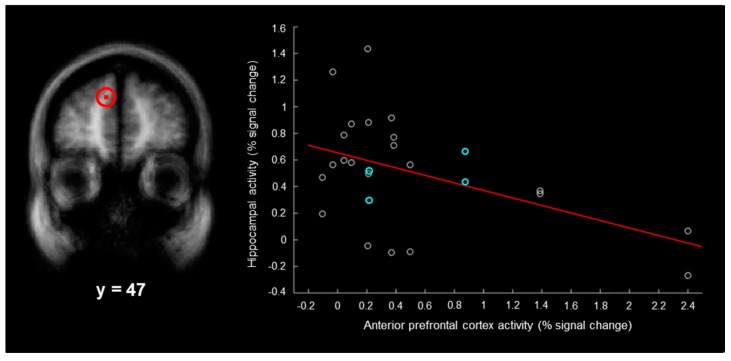
Relationship between the magnitude of spatial memory activity in the left anterior prefrontal cortex and the hippocampus. **Left**, left anterior prefrontal cortex activity associated with left-hits and left-misses (circled in red; coronal view). **Right**, for each participant, the magnitude of hippocampal activity associated with false memories as a function of the magnitude of left anterior prefrontal cortex activity associated with false memories (the best-fit line is shown in red; results from male participants are shown in blue).

The contrast of left-hits and left-misses also produced three activations in the left anterior/dorsolateral prefrontal cortex (*x* = −21, *y* = 35, *z* = 37, Brodmann area (BA) 9 within the superior frontal sulcus, size = 27 mm^3^; *x* = −9, *y* = 47, *z* = 28, BA9 within the anterior prefrontal cortex, size = 27 mm^3^; *x* = −36, *y* = 41, *z* = 10, BA46 within the inferior frontal sulcus, size = 27 mm^3^). For each participant, we plotted the magnitude of activity associated with right–“left”–“very sure” responses in each of the two hippocampal regions as a function of the magnitude of activity in each anterior/dorsolateral prefrontal cortex region. For the anterior prefrontal cortex region (Figure 3, left), there was a significant negative correlation between the magnitude of activity in the hippocampus and the magnitude of activity in this region (Figure 3, right; *r* = −0.48, *p* < 0.05, Bonferroni corrected for the three hippocampal-prefrontal cortex correlations). Although the present results were not powered to assess gender effects, the correlation was nearly identical (*r* = −0.50, *p* < 0.05) after removing males from the analysis. It should be highlighted that all of the activations evaluated were associated with “very sure” responses (i.e., confidence was held constant); thus, the activations were not correlated with confidence. One limitation of the current study is that our sample size was relatively small (i.e., the study was underpowered); however, this would be expected to produce null results. As significant results were observed, the sample size was not of major concern.

We conducted additional analyses to assess whether the negative correlation between activity in the anterior dorsolateral prefrontal cortex and the hippocampus was specific to false memories (i.e., right–“left”–“very sure” responses). The correlation between these regions was not significant for either right–“left”–“unsure” responses (*r* = −0.20, *p* > 0.20) or right–“right”–“very sure” responses (*r* = −0.13, *p* > 0.20). These findings indicate that the anterior dorsolateral prefrontal cortex and the hippocampus were not correlated during the analogous low confidence responses or during confident true memories.

To determine whether there were behavioral differences between participants with hippocampal based false memories and participants with anterior dorsolateral prefrontal cortex based false memories, we conducted a post hoc split-half analysis. The behavioral performance of the participants with higher magnitudes of anterior prefrontal cortex activity was compared with the behavioral performance of the participants with lower magnitudes of anterior prefrontal cortex activity (the participant with an intermediate magnitude of activity was left out such that there were equal numbers in each group). There was no difference between these groups of participants in either overall behavioral accuracy (*t*(11) < 1) or the rate of false memories for items in the “left” visual field (i.e., right–“left”–“very sure” responses/all right–“very sure” responses; *t*(11) < 1).

## 4. Discussion

In the present study, we found that the magnitude of activity in the hippocampus was negatively correlated with the magnitude of activity in the left anterior prefrontal cortex during false memories. These findings suggest that false memories may be mediated by the hippocampus (and not the anterior prefrontal cortex) in some participants and the anterior prefrontal cortex (and not the hippocampus) in other participants. This is the first time, to our knowledge, that participants have been shown to engage either the hippocampus or the anterior prefrontal cortex during false memories.

It is notable that the contrast of left-hits and left-misses only produced activations in the left hippocampus, which replicates a previous study [28]. As the left hippocampus has been associated with verbal memory [30,31], these activations might have reflected language processing associated with accurate memory for the spatial location of each shape (i.e., the verbal label “left”). Alternatively, the hemispheric laterality in the hippocampus may have been a consequence of limited power.

The current findings may shed light on the variable nature of false memory activity previously reported in the hippocampus [2,3,32]. In the present study, the magnitudes of hippocampal false memory activity ranged from −0.27 to 1.43 percent across participants, the magnitudes of left anterior false memory activity ranged from −0.10 to 2.40 percent, and there was a negative correlation between these regions (Figure 3, right). This demonstrates that false memories were only based on hippocampal activity in some participants and were only based on left anterior prefrontal cortex activity in other participants. If some groups of participants engage the anterior prefrontal cortex and not the hippocampus during false memories, this would predict relatively low magnitudes of hippocampal activity during false memory, which has previously been observed [2,8,9]. On the other hand, if some groups of participants engage the hippocampus and not the anterior prefrontal cortex, this would predict relatively high magnitudes of hippocampal activity during false memory, which has also been observed [4,5,6,10]. Future research will be needed to determine the specific stimulus or task conditions under which the hippocampus and the anterior prefrontal cortex are more or less strongly associated with false memory.

The present negative correlation between the magnitude of activity in the hippocampus and the magnitude of activity in the anterior prefrontal cortex suggests that these regions interact during false memories. One possibility is that, during false memories, the left anterior/dorsolateral prefrontal cortex may be activated due to (incorrect) context memory or high confidence and this region may inhibit the hippocampus to reduce the amount of potentially conflicting information. (note that it is also possible that another region of the dorsolateral prefrontal cortex, such as the left inferior dorsolateral prefrontal cortex (BA46) activation in the present study, may reflect language processing, which can also give rise to false memories [33,34]). Conversely, as correlation does not confer directionality, the hippocampus may be activated due to (incorrect) binding and this region may inhibit the anterior/dorsolateral prefrontal cortex to reduce the amount of potentially conflicting information. Although the direction of the interaction between the anterior/dorsolateral prefrontal cortex and the hippocampus is uncertain, the anterior/dorsolateral prefrontal cortex has been generally associated with inhibition [21,22], and thus it is likely that this region inhibited the hippocampus during false memories.

Single-cell recording evidence in non-human animals also indicates that the prefrontal cortex and the hippocampus interact during memory for item and context information [35]. For instance, a recent study in rats demonstrated that information flowed from the hippocampus to the prefrontal cortex during item-in-context memory encoding and information flowed from the prefrontal cortex to the hippocampus during item-in-context memory retrieval [36]. As the present findings were observed during retrieval, these behavioral neuroscience findings provide additional evidence that, for some participants, the anterior prefrontal cortex inhibited the hippocampus during false memories. Future studies could employ simultaneous depth electrode recording in the hippocampus, such as in patients with intractable epilepsy, and scalp electrophysiological recording to investigate the nature of the interactions between these regions during false memory construction.

## 5. Conclusions

In the present study, we found a negative correlation between the magnitudes of activity in the anterior prefrontal cortex and the hippocampus. This suggests that the anterior prefrontal cortex might inhibit the hippocampus during false memories and that participants engage either the anterior prefrontal cortex or the hippocampus during false memories.
